# New host, geographic records, and histopathologic studies of *Angiostrongylus* spp (Nematoda: Angiostrongylidae) in rodents from Argentina with updated summary of records from rodent hosts and host specificity assessment

**DOI:** 10.1590/0074-02760150371

**Published:** 2016-03

**Authors:** María del Rosario Robles, John M Kinsella, Carlos Galliari, Graciela T Navone

**Affiliations:** 1 Centro Científico Tecnológico-Consejo Nacional de Investigaciones Científicas y Técnicas, Centro de Estudios Parasitológicos y de Vectores, Buenos Aires, Argentina; 2 HelmWest Laboratory, Missoula, MT, USA

**Keywords:** Angiostrongylus, histopathology, host specificity, rodents, Sigmodontinae, Argentina

## Abstract

To date, 21 species of the genus *Angiostrongylus* (Nematoda: Angiostrongylidae) have been reported around the world, 15 of which are parasites of rodents. In this study, new host, geographic records, and histopathologic studies of *Angiostrongylus* spp in sigmodontine rodents from Argentina, with an updated summary of records from rodent hosts and host specificity assessment, are provided. Records of *Angiostrongylus costaricensis* from *Akodon montensis* and*Angiostrongylus morerai* from six new hosts and geographical localities in Argentina are reported. The gross and histopathologic changes in the lungs of the host species due to angiostrongylosis are described. Published records of the genus *Angiostrongylus* from rodents and patterns of host specificity are presented. Individual *Angiostrongylus*species parasitise between one-19 different host species. The most frequent values of the specificity index (STD) were between 1-5.97. The elevated number of host species (n = 7) of *A. morerai* with a STD = 1.86 is a reflection of multiple systematic studies of parasites from sigmodontine rodents in the area of Cuenca del Plata, Argentina, showing that an increase in sampling effort can result in new findings. The combination of low host specificity and a wide geographic distribution of *Angiostrongylus* spp indicates a troubling epidemiological scenario although, as yet, no human cases have been reported.

The main definitive hosts of angiostrongylid nematodes of the superfamily Metastrongylidoidea are carnivores and rodents and the known intermediate hosts are molluscs (e.g., Acha & Szyfres 2003). To date, 21 species of the genus *Angiostrongylus* Kamensky 1905 have been reported around the world. Six species have been described infecting carnivores:*Angiostrongylus vasorum* Baillet 1866, *Angiostrongylus raillieti* Travassos 1927, *Angiostrongylus gubernaculatus* Dougherty 1946,*Angiostrongylus chabaudi* Biocca 1957, *Angiostrongylus daskalovi* Yanchev & Genov 1988, a, and *Angiostrongylus felineus* Vieira et al. 2013, a, and the remainder from rodents: *Angiostrongylus tateronae* Baylis 1928, *Angiostrongylus cantonensis* (Chen 1935),*Angiostrongylus sciuri* Merdevenci 1964, *Angiostrongylus mackerrasae* Bhaibulaya 1968,*Angiostrongylus sandarsae* Alicata 1968, *Angiostrongylus petrovi* Tarzhimanova & Chertkova 1969, *Angiostrongylus dujardini* Drozdz & Doby 1970,*Angiostrongylus schmidti* Kinsella 1971, *Angiostrongylus costaricensis* Morera & Céspedes 1971, *Angiostrongylus malaysiensis* Bhaibulay & Cross 1971, *Angiostrongylus ryjikovi* (Jushkov 1971),*Angiostrongylus andersoni* (Petter 1972), *Angiostrongylus siamensis* Ohbayashi, Kamiya & Bhaibulaya 1979, *Angiostrongylus morerai* Robles, Navone & Kinsella, 2008, and *Angiostrongylus lenzii* Souza et al. 2009 (Baillet 1866, Travassos 1927, Baylis 1928, Dougherty 1946, Mackerras & Sandars 1955, Biocca 1957, Merdivenci 1964, Alicata 1968, Bhaibulaya 1968, Tarzhimanova & Chertkova 1969, Bhaibulaya & Cross 1971, Doby et al. 1971, Jushkov 1971, Kinsella 1971, Morera & Céspedes 1971, Petter 1972, Ohbayashi et al. 1979, Yanchev & Genov 1988, Robles et al. 2008, Souza et al. 2009, Vieira et al. 2013, Spratt 2015). Except for two species, *A. costaricensis* and*A. siamensis,* which infect the mesenteric arteries of the caecum, all species inhabit the pulmonary arteries and right ventricle of the heart.

Among rodents, species of *Angiostrongylus* are distributed in the Cricetidae, Echimyidae, Gliridae, He- teromyidae, Muridae, and Sciuridae. The best studied and most widely distributed species are *A. cantonensis* and*A. costaricensis,* which are primarily parasites of rodents but carnivores, marsupials and primates have also been recorded as definitive hosts (Maldonado et al. 2012) as well as abnormal/aberrant hosts (Spratt 2015). Both species are recognised as zoonotic; the first is the cause of the disease eosinophilic meningoencephalitis from different continents and the second of abdominal angiostrongyliasis from the Americas (Acha & Szyfres 2003, Maldonado et al. 2012, Spratt 2015).

The life cycles of eight species parasitising rodents have been studied: *A. andersoni*, *A. cantonensis, A. costaricensis, A. dujardini, A. mackerrasae, A. malaysiensis, A. siamensis,* and *A. schmidti*. In those species inhabiting the pulmonary arteries, eggs deposited by adults develop to first stage larvae in the lungs which move up the airways, are swallowed and pass in the faeces. This developmental pathway is exempliﬁed by *A. andersoni*, *A. dujardini*, and *A. schmidti* (Kinsella 1971, Bhaibulaya 1975, Mota & Lenzi 2005, Spratt 2015). The resultant pathology has been described in species such as *A. cantonensis*, *A. costaricensis*, *A. mackerrasae*, *A. morerai*, *A. sandarsae*,*A. schmidti*, and *A. siamensis* (Mackerras & Sandars 1955, Alicata 1968, Kinsella 1971,Tesh et al. 1973, Ohbayashi et al. 1979, Mota & Lenzi 2005, Robles et al. 2012).

One of the most important properties characterising a parasite taxon is its host specificity. It is indicative of intrinsic biological characteristics of both host and parasite and an emergent property of their ecological and evolutionary relationship (Dick & Patterson 2007). Host specificity can be defined as the extent to which a parasite taxon is restricted in the number of host species used at a given stage in the life cycle (Poulin 2007).

In this paper, we provide new host and geographical records for two species of*Angiostrongylus* from sigmodontine rodents in Argentina and describe the gross and histopathologic changes in the lungs of the host species due to angiostrongylosis. Moreover, we present comprehensive data on all the records of the genus *Angiostrongylus* from rodents and evaluate patterns of host specificity.

## MATERIALS AND METHODS

Cricetid rodents were trapped during different field studies between 2007-2012 (see acknowledgements and financial support) and the following species were examined for angiostrongylid nematodes: eight specimens of *Deltamys kempi* Thomas 1917 from Reserva Natural de la Costanera Sur (34º36’S 58º27’W), Ciudad Autónoma de Buenos Aires and La Balandra (34º56’S 57º42’W), Partido de Berisso, province of Buenos Aires, 11 specimens of *Akodon montensis* Thomas 1913 from RP2, 6 km NE, Arroyo Paraíso, (27º12’47.7”S 54º01’59.9”W) and Salto El Paraíso, Arroyo Paraíso (27º13’49.8”S 54º02’24.3”), department of Guaraní, 27 *A. montensis* and three *Sooretamys angouya* (Fischer 1814) from Refugio Moconá (27°8’29.01”S 53°55’40.44”W), department of San Pedro, province of Misiones, 16 *Akodon azarae bibianae* Massoia 1971, 10*Calomys callosus* Rengger 1830, and three *Necromys lasiurus liciae* Contreras 1982 from Reserva El Bagual (26º18’12.81”S 58º48’51.57”W), department of Laishi and Estación de Animales Silvestres Guaycolec, Ruta Nacional 11, km 1201 (25º58’40.65”S 58º09’49.82”), department of Formosa, province of Formosa.

The viscera (included lungs) were fixed whole in 10% buffered formalin and examined. Pulmonary arteries and veins were opened and observed for adult worms using a stereoscopic microscope. Adult nematodes were collected, preserved in 70% ethanol, cleared in lactophenol, and studied under a light microscope. Drawings were made with the aid of a drawing tube. Each of the five lobes of the lungs was trimmed in the subterminal transversal part, processed, sectioned at 5 µm (± 25 sections per slide), stained with haematoxylin and eosin (H&E), and examined microscopically.

Quantitative parameters of prevalence (P = specimens parasitised/specimens examined x 100) was calculated according to Bush et al. (1997) for each host species and locality.

Records of species of *Angiostrongylus* from rodents were compiled from the literature (scientific papers and book sections). When necessary, scientific names of mammal hosts have been updated following Edwards et al. (1993), Wilson and Reeder (2005), Weksler et al. (2006), and Srinivasulu and Srinivasulu (2011). In order to evaluate host specificity, the specificity index (S_TD_) by Poulin and Mouillot (2003) was calculated. This index measures the average taxonomic distinctness of all host species used by a parasite species. All mammal species included were ﬁtted into a taxonomic structure with six hierarchical levels above species, i.e., genus, subfamily, family, superfamily, order, and class (Mammalia). The range of index can vary between 1-6, and since the index cannot be computed for parasites exploiting a single host species, the value of zero is assigned to reﬂect strict host speciﬁcity. The value of this index is inversely proportional to host specificity. The asymmetries in the taxonomic distribution of host species were calculated through variance in taxonomic distinctness (VarS_TD_) (Poulin & Mouillot 2003). A record was defined as the finding of a parasite species on a definitive host and, at a given locality, regardless of the number of host sampled and of nematodes collected on a particular host. The aberrant host species reported (Maldonado et al. 2012, Spratt 2015) which showed signs of disease were included in the calculation of host speciﬁcity, but not the experimentally infected or accidental host species.

Adult specimens and H&E stained sections (slides) of lung were deposited in the Helminthological Collection of the Museo de La Plata (CHMLP *A. costaricensis* 7052 and *A. morerai* 7053-7059, respectively) and the hosts were deposited in the Mastozoological Collections of the Centro Nacional Patagónico (CNP 1968, 2338, 3004, 3723, 4079, 4080, 4027, 4602, field number CG 70, 78, RR 33), Puerto Madryn, Chubut, Argentina.

*Ethics* - The research has been conducted according to Argentine laws. Sample collection was carried out during fieldwork under official permits granted by Fauna and Flora of the Province of Buenos Aires (expedient 22500-7981/10), Ministry of Industry and Environment of the Province of Formosa (authorisation n/n; transit guide: 004076), Ministry of Ecology, Renewable Natural Resources, and Tourism of Misiones (authorisation 24 and 27, transit guides: 000316 and 000371). This study was carried out in accordance with the recommendations in the Guide for the Care and Use of Laboratory Animals of the National Institutes of Health. The specimens obtained with methods for live capture were studied and humanely sacrificed following the procedures and protocols approved by national laws (Animal Protection National law 14.346 and references in the provincial permits) and Ethical Committee for Research on Laboratory Animals, Farm, and Obtained from Nature of National Council of Scientific and Technical Research (resolution 1047, section 2, annex II), and subsequently by National Agency for the Promotion of Science and Technology of Argentina (PICT 2010-0924). No endangered species were involved in this study.

## RESULTS

**Fig. 1:**
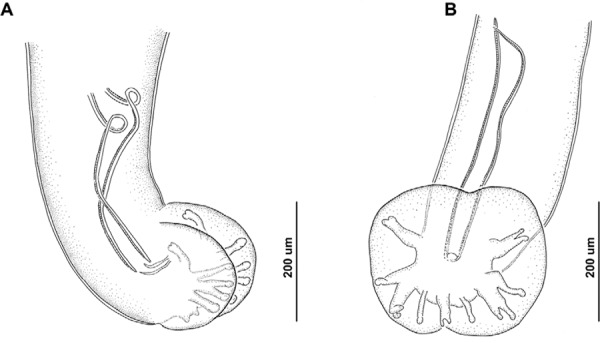
: male adult specimens of *Angiostrongylus* spp. A:*Angiostrongylus costaricensis* from caecal mesenteric arteries of *Akodon montensis* of the province of Misiones, medium lateral view; B: *Angiostrongylus morerai* from the lungs and heart from *Calomys callosus* of the province of Formosa, ventral view.

A single male specimen of *Angiostrongylus* found in the caecal mesenteric arteries of *A. montensis* from El Soberbio was identified as *A. costaricensis.* Adult specimens found in the pulmonary arteries and heart of *D. kempi* from La Balandra and Reserva Natural de la Costanera Sur, *A. montensis* and *S. angouya*from Refugio Mocona, and *A. azarae*, *C. callosus*, and *N. lasiurus* from Reserva El Bagual were identified on the basis of the morphology of the bursa, spicules, and diagnostic measurements as *A. morerai* (Fig. 1).

Table I lists prevalence of infections for all hosts examined. The prevalence of *A. costaricensis* was very low (9%). The highest prevalence of *A. morerai* was recorded in*D. kempi*. The region with the most records of this nematode was La Pampa ecoregion (P = 62.5%) in the province of Buenos Aires. The Selva Paranaense (province of Misiones) and Chaco Húmedo (province of Formosa) ecoregions showed similar values (P = 20% and 17.4%, respectively) (Table I).

**TABLE I:** Prevalence of *Angiostrongylus* spp for each host species and locality

Host species	Locality	Prevalence by population (%)	Prevalence by ecoregion (%)
*Angiostrongylus costaricensis*			
*Akodon montensis*	Arroyo Paraíso	1/11 (9)	Selva Paranaense (9)

*Angiostrongylus morerai*			
*Deltamys kempi*	La Balandra	1/4 (25)	La Pampa (62.5)
	Reserva Natural de la Costanera Sur	4/4 (100)	
*A. montensis*	Refugio Moconá	4/27 (14.8)	Selva Paranaense (20)
*Sooretamys angouya*	Refugio Moconá	2/3 (66)	
*Akodon azarae bibianae*	Reserva El Bagual	2/11 (18.18)	Chaco Húmedo (17.4)
*Calomys callosus*	Reserva El Bagual	1/10 (10)	
*Necromys lasiurus liciae*	Reserva El Bagual	1/2 (50)	

**Fig. 2:**
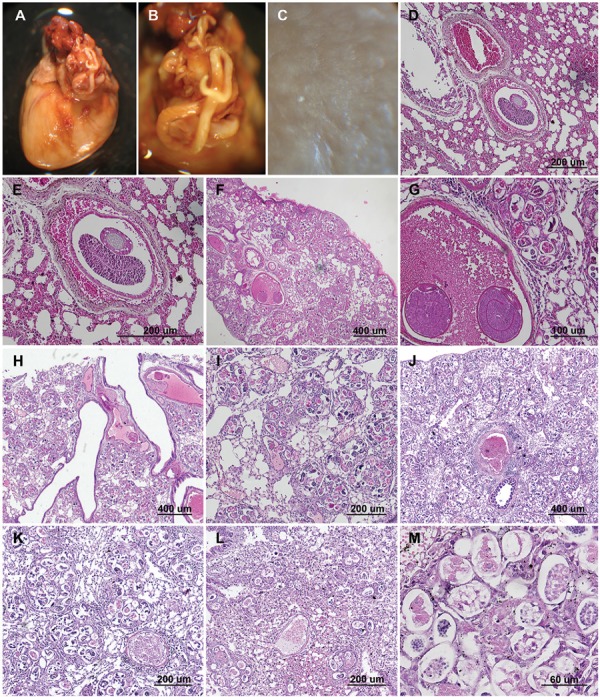
: macroscopic and histopathological examination of heart and lung infected with *Angiostrongylus morerai*. A: adult specimen in pulmonary artery of *Akodon azarae*; B: detail of female specimen in pulmonary artery; C: lung with macroscopic lesions (ﬁrm nodules) of verminous pneumonia; D: lungs of*Deltamys kempi* with detail of the interior of a blood vessel containing adult worm; E: detail of adult worm; F: lungs of*Akodon montensis* with superficial interstitium and alveoli containing eggs, larvae, and adult worm; G: detail of eggs, larvae, and adult worm in interior of a blood vessel; H: lungs of*A. azarae* with detail of interstitium, alveoli, and vessels contained nematode eggs and larvae; I: detail of granulomatous inﬂammatory reactions, vessel, and interstitium contained eggs and nematode larvae; J: lungs of *Calomys callosus* with superﬁcial interstitium and alveoli containing nematode larvae; K: detail of granulomatous inﬂammatory reactions surround each set of eggs and larvae; L: lungs of *Necromys lasiurus* with interstitium, alveoli, and vessels contained nematode eggs and larvae; M: granulomatous inﬂammatory reactions, vessel, and interstitium contained eggs on different embryonic stages and nematode larvae.

Specimens of *A. morerai* were present in heart chambers (Fig. 2A) and in pulmonary arteries sometimes showing the complete obliteration of the lumen (Fig. 2B). The infected rodents showed macroscopic lesions (firm nodules) of verminous pneumonia in three, four, or five lobes. Each lung lobe contained multiple small yellowish nodules scattered throughout the parenchyma (Fig. 2C).

Additionally, histopathology examination of tissue fragments showed multiple nodules in the vessels, interstitium, and alveoli. Nodules were formed by larvae surrounded by an elevated number of granulocyte and mononuclear cells (Fig. 2M). The vessels, interstitium, and alveoli contained nematode larvae with mild to moderate interstitial fibrosis (Fig. 2F-M). Worms were approximately 80-200 µm long and contained numerous discrete basophilic and eosinophilic granules (Fig. M). Numerous nodules (set of eggs and larvae) surround by granulomatous reactions were situated under the pleural surface (Fig. 2F, J). Several damaged capillaries and small arterioles were observed (Fig. 2D-H).

The lobe with the greatest intensity of larvae proportionally was the left upper followed by the right lower and right medium lobes, the right upper and left lower lobes, had similar, but smaller, intensities of infection. As estimation about one-five larvae per 200 µm 2 x 5 µm thickness could be observed in the left upper lobe. In the other lobes, the nodules were more scattered. The host with the most nodules (set of larvae) surrounded by granulomatous reactions was *C. callosus* (Fig. 2J, K).

**Fig. 3:**
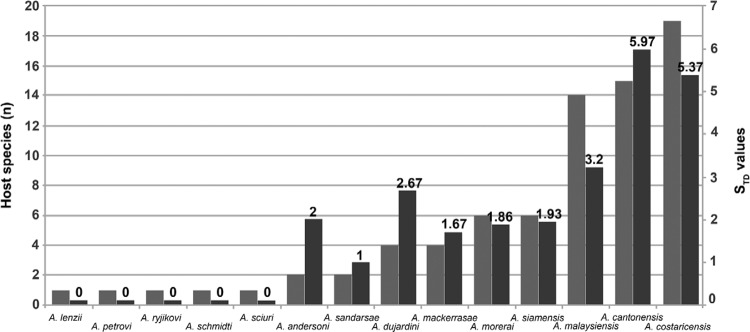
: number of host species (left) and host specificity values (right) for *Angiostrongylus* species from rodents. STD: specificity index.

Number of host species for all *Angiostrongylus* species found in rodents and values of S_TD_ and VarS_TD_ for each species are shown in Table II and depicted in Fig. 3. The distribution of number of host species was skewed considering only the natural infection by angiostrongylosis (Fig. 3). The figures clearly show that most *Angi- ostrongylus* species parasitise between one-19 different host species: five *Angiostrongylus* species were associated with a single species, *A. andersoni* and *A. sandarsae* were found in two host species, *A. dujardini* and *A. ma- ckerrasae* in four host species, *A. morerai* and*A. siamensis* in six host species, and the rest in more than 10 host species. The values of S_TD_ were between 1-5.97. The value of zero was assigned for five species to reﬂect the strict host speciﬁcity. *A. andersoni*, *A. sandarsae*, *A. mackerrasae*, *A. morerai*, and *A. siamensis* parasitise species hosts that belong to different subfamilies (S_TD_ = 1-2), *A. dujardini* to different families (S_TD_ = 2-3), *A. malaysiensis* to different superfamilies (S_TD_ = 3-4), and *A. cantonensis* and*A. costaricensis* to different orders (S_TD_ = 5-6).

**TABLE II:** List of *Angiostrongylus* spp from mainly rodents (and no rodents) with values of host specificity (STD) and variance (VarSTD) taxonomic distinctness index

Parasite species	Host species	S_TD_ and VarS_TD_	Country	Site infection	References
*Angiostrongylus andersoni* (Petter 1972)	*Gerbilliscus kempi* ^*a*^ *Taterillus gracilis* ^*b*^	2 and 0	Upper Volta (Africa)	Large abscesses in the lungs	Petter (1972)
*Angiostrongylus cantonensis* (Chen 1935)	*Bandicota indica Diplothrix legata Melomys burtoni Melomys cervinipes Podomys floridanus* ^*c*^ *Rattus rattus Rattus norvegicus* No rodents *Canis lupus familiaris Didelphis virginiana Equus caballus Homo sapiens Pteropus poliocephalus Pteropus alecto Suncus murinus Varecia variegata*	5.97 and 3.55	China (Asia); Asian and Pacific Islands and Australia (Oceania); Brazil, Cuba, Haiti, Jamaica, Puerto Rico, United States of America (USA) (America)	Lungs and heart (central nervous system)	Chen (1935), Mackerras & Sandars (1955), Cross (1979), Aguiar et al. (1981), Andersen et al. (1986), Alicata (1988), Wright et al. (1991), Cooke-Yarborough et al. (1999), Barrett et al. (2002), Kim et al. (2002), Lindo et al. (2002), Raccurt et al. (2003), Smales et al. (2004), Simões et al. (2011), Lunn et al. (2012), Maldonado et al. (2012), Ma et al. (2013), Morton et al. (2013), Okano et al. (2014)
*Angiostrongylus costaricensis*Morera & Céspedes 1971	*Akodon montensis Liomys adspersus Melanomys caliginosus* ^*d*^ *Oligoryzomys fulvescens* ^*e*^ *Oligoryzomys nigripes Oxymycterus hispidus* ^*f*^ *Nephelomys albigularis* ^*g*^ *Proechimys*sp. *R. rattus R. norvegicus Sigmodon hispidus Sooretamys angouya* ^*h*^ *Zygodontomys revicaudas* ^*i*^ No rodents*Nasua narica Saguinus mystax Symphalangus syndactylus* ^*j*^ *Aotus nancymaae Procyon lotor D. virginiana*	5.37 and 4.28	Brazil, Colombia, Costa Rica, Dominican Republic, Ecuador, Mexico, Panama, Peru, Puerto Rico, USA, Venezuela, Argentina (America)	Caecum mesenteric arteries (intestinal wall)	Morera (1970), Tesh et al. (1973), Monge et al. (1978), Ubelaker & Hall (1979), Malek (1981), Andersen et al. (1986), Teixeira et al. (1990), Vargas et al. (1992), Juminer et al. (1993), Miller et al. (2006), Maldonado et al. (2012)
*Angiostrongylus dujardini* Drozdz & Doby 1970	*Apodemus sylvaticus Apodemus flavicollis Microtus subterranus* ^*k*^ *Myodes glareolus* ^*l*^	2.67 and 0.22	France, Portugal, Hungary, Finland (Europe)	Lungs and heart	Dróźdź & Doby (1970), Doby et al. (1971), Mészáros (1972), Tenora et al. (1983)
*Angiostrongylus lenzii* Souza et al. 2009	*A. montensis*	0	Brazil (America)	Lungs and heart	Souza et al. (2009)
*Angiostrongylus mackerrasae*Bhaibulaya 1968	*Rattus fuscipes Rattus lutreolus R. norvegicus*	1.67 and 0.22	Queensland, New South Wales, Tasmania (Oceania)	Lungs and heart (central nervous system)	Bhaibulaya (1968, 1975), 1975), Stokes et al. (2007)
*Angiostrongylus malaysiensis*Bhaibulaya & Cross 1971	*Berylmys bowersi* ^*m*^ *Leopoldamys sabanus* ^*n*^ *Maxomys surifer* ^*o*^ *Maxomys whiteheadi* ^*j*^ *Niviventer cremoriventer* ^*p*^ *Rattus annandalei Rattus argentiventer Rattus exulans R. norvegicus Rattus rattus diardii Rattus tiomanicus Sundamys muelleri* ^*q*^ No rodents*S. murinus Tupaia glis*	3.2 and 5.61	Malaysia, Indonesia, Thailand (Asia-Oceania)	Lungs and heart (central nervous system)	Bhaibulaya & Cross (1971), Bhaibulaya & Techasophonmani (1972), Carney & Stafford (1979), Cross (1979), Lim & Ramachandran (1979), Pipitgool et al. (1997)
*Angiostrongylus morerai* Robles, Navone & Kinsella 2008	*Akodon azarae Akodon dolores Apodemus montensis Calomys callosus Deltamys kempi Necromys lasiurus S. angouya*	1.86 and 0.12	Argentina (America)	Lungs and heart	Robles et al. (2008, 2012), 2012)
*Angiostrongylus petrovi*(Tarzhimanova & Chertkova 1969)	*Dryomys nitedula*	0	Azerbaidzhan (Asia)	Lungs and heart	Tarzhimanova & Chertkova (1969), Spratt (2015)
*Angiostrongylus ryjikovi* Jushkov 1971	*Myodes rutilus* ^*q*^	0	Soviet Union (Eurasia)	Lungs and heart	Jushkov (1971), Spratt (2015)
*Angiostrongylus sandarsae* Alicata 1968	*Gerbil tatera* ^*r*^ *Mastomys natalensis* ^*s*^	0-1	Mozambique, Kenya (Africa)	Lungs and heart	Alicata (1968), Kamiya & Fukumoto (1988), Spratt (2015)
*Angiostrongylus schmidti* Kinsella 1971	*Oryzomys palustris*	0	USA (America)	Lungs and heart	Kinsella (1971)
*Angiostrongylus sciuri* Merdevenci 1964	*Sciurus vulgaris*	0	Turkey (Eurasia)	Lungs and heart	Merdivenci (1964), Spratt (2015)
*Angiostrongylus siamensis*Ohbayashi, Kamiya & Bhaibulaya 1979	*B. indica Bandicota savilei Berylmys berdmorei* ^*t*^ *L. sabanus* ^*n*^ *Maxomys surifer* ^*o*^ *R. rattus*	1.93 and 0.06	Thailand (Asia)	Mesenteric arteries	Ohbayashi et al. (1979, 1983), 1983), Kamiya et al. (1980)
*Angiostrongylus tateronae* Baylis 1928	*Apodemus mystacinus*	0	Nigeria (Africa), Albania (Europa)	Lungs and heart	Baylis (1928), Spratt (2015)

*a*: *Tatera kempi*;*b*: *Taterillus nigeriae*;*c*: *Neotoma floridanus*;*d*: *Oryzomys caliginosus*;*e*: *Oryzomys fulvescens*;*f*: *Oxymycterus judex*;*g*: *Oryzomys albigularis*;*h*: *Oryzomys ratticeps*;*i*: *Zygodontomys microtinus*;*j*: *Hylobates syndactylus*,*Maxomys whiteheadi*; *k*:*Pitymys subterranus*; *l*:*Clethrionomys glareolus*; *m*:*Rattus bowersi*; *n*:*Rattus sabanus*; *o*:*Maxomys surifer*; *p*:*Rattus cremoriventer*; *q*:*Clethrionomys rutilus*, *Rattus muelleri*; *r*:*Indeterminate*; *s*:*Praomys natalensis*; *t*:*Rattus berdmorei.*

## DISCUSSION

This is the first record of *A. morerai* from *A. montensis*, *C. callosus*, *D. kempi*,*N. lasiurus*, and *S. angouya* expanding its geographic distribution to the south and northwest of the provinces of Buenos Aires, Misiones, and Formosa. *A. costaricensis* is recorded for first time in *A. montensis* and in Argentina. The presence of*Angiostrongylus* spp in these hosts indicates the ingestion of unknown intermediate hosts, which are apparently frequent in the diet of rodents of the tribe Akodontini (e.g., *Akodon*, *Necromys*,*Deltamys*).

With respect to the gross and histopathologic changes in the lungs of the host species, a different degree of pathogenicity was observed among the hosts, with the highest being in *C. callosus* (Phyllotini). This is the first record of *Angiostrongylus* sp. in this tribe of sigmodontine rodents. As demonstrated in Robles et al. (2012), in*A. morerai*, the resulting immune reaction can cause interstitial fibrosis and the destruction of small capillaries and arterioles. In that study and here, extensive lesions were apparently caused by a single male and female (Fig. 2A, B). Macroscopic lesions of verminous pneumonia in the lungs were similar to those described for *A. mackerrasae* by Mackerras and Sandars (1955) and *A. sandarsae* by Alicata (1968). Histopathological examination revealed nodules formed as a result of larvae being surrounded by granulocytes and mononuclear cells (Fig. 2C-M).

Of the 15 species of *Angiostrongylus* parasitic in rodents, detailed descriptions of histopathologic changes are available for seven (*A. cantonensis*, *A. costaricensis*, *A. mackerrasae*, *A. morerai*, *A. sandarsae*, *A. siamensis*, and *A. schmidti*) and the life cycles of eight species have been studied (*A. andersoni*, *A. cantonensis, A. costaricensis, A. dujardini, A. mackerrasae, A. malaysiensis, A. siamensis*, and *A. schmidti*) (Mackerras & Sandars 1955, Alicata 1968, Kinsella 1971, Bhaibulaya 1975, Mota & Lenzi 2005, Robles at al. 2012). However, to know the complete pathogenicity and potential transmission of each parasitic species, studies on intermediate hosts and the reaction of the larvae in the affected organs must be completed.

The presence of *A. morerai* in Argentina in different ecoregions indicates that environmental features may have little influence on geographic distribution, although it is interesting to note that apparently these can influence frequency and abundance. Prevalence in the La Pampa ecoregion was considerably higher than Selva Paranaense (20%) and Chaco Húmedo (17.4%). The question is whether the differences in the frequency and abundance of *Angiostrongylus*spp may be due to the sampling effort and/or to the distribution of definitive and intermediate hosts and/or to the susceptibility of both. For example, with respect to the latter, Combes (2001) proposed different filters of parasite-host association; encounter filters (biodiversity and behaviour) and compatibility filters (resource and density).

Five species of *Angiostrongylus* have been reported in North and South American rodents: *A. cantonensis*, *A. costaricensis*, *A. lenzii, A. morerai*, and *A. schmidti*. Notably, the snails *Achatina fulica* (Bowdich 1822), *Pomacea caniculata* (Lamarck 1828), *Phyllocaulis variegatus* (Semper 1885), *Phyllocaulis soleiformis*(Orbigny 1835), *Belocaulus angustipes* (Heynemann 1885) are recorded from the area studied in this survey, and have all been previously recorded as intermediate hosts of *A. cantonensis* and/or *A. costaricensis* (Teixeira et al. 1993, Díaz et al. 2013, Gregoric et al. 2013). Accordingly, the low host specificity of these *Angiostrongylus* spp it is puzzling that there been no cases of eosinophilic meningoencephalitis or abdominal angiostrongylosis in Argentina to date. Spratt (2015) partly answer to a similar situation, since the reports in the literature of many species of *Angiostrongylus* in rodents reﬂect lack of opportunity or interest in examining nonurban and nonagricultural hosts (Table II).

The elevated number of host species (n = 7) of *A. morerai* with a S_TD_= 1.86 is a reflection of multiple systematic studies of parasites from sigmodontine rodents in the area of Cuenca del Plata Argentina, showing that an increase in sampling effort can result in new findings. Therefore, a low number of host species used by other *Angiostrongylus* species may be an artifact caused by lack of sampling effort.

This is the first attempt to describe general patterns of host specificity of*Angiostrongylus* from rodents through a quantitative approach. Host specificity values did not include the hosts recorded as part of experimental infections or accidental hosts (Table II). Those hosts include *Taterillus* cf. *congicus* for*A. andersoni*, *Aepyprymnus rufescens* and*Macropus rufogriseus* for *A. cantonensis*,*Pteropus alecto* for *A. mackerrasae*,*Meriones unguiculatus*, *Mesocricetus auratus*,*Mus musculus*, *Peromyscus leucopus*,*Rattus norvegicus*, and *Sigmodon hispidus* for*A. schmidti* (Kinsella 1971, Petter & Cassone 1975,McKenzie et al. 1978, Higgins et al. 1997, Barrett et al. 2002). However, those studies support the conclusions of this survey, since the addition of hosts from other families and orders would only increase the S_TD_ values.

In conclusion, the distribution of *Angiostrongylus* spp shows no environmental limits, demonstrates low host specificity, and indicates that their host range has probably been underestimated. In addition, there are other host records of some species of *Angiostrongylus* which need to be confirmed by morphological and molecular analysis (Robles et al. 2012). Moreover, it is necessary to explore the different degrees of pathogenicity in various hosts, mainly in those cases that are phylogenetically more distant (different host family) to analyse which are the filters (meeting, immunological, etc.) that determine host distribution. These results would allow anticipating contingencies and prevention planning for diseases caused by angiostrongylosis.

There is a need to increase awareness in the human population about the risk of contracting angiostrongyliasis and healthcare providers should consider these parasites on the South American continent when making medical diagnoses. Moreover, surveillance and control of intermediate and definitive hosts as well as health education should be done to avoid human infections.
